# Suicide presentation and the risk at the time of the mandatory quarantine for the SARS-Cov-2 pandemic: medico-legal and forensic aspects

**DOI:** 10.1192/j.eurpsy.2022.1356

**Published:** 2022-09-01

**Authors:** C. Scalise, M.A. Sacco, A. Zibetti, P. De Fazio, P. Ricci, I. Aquila

**Affiliations:** 1 Institute of Legal Medicine, Medical And Surgical Science, Catanzaro, Italy; 2 Institute of Psichiatry, Medical And Surgical Sciences, Catanzaro, Italy

**Keywords:** Suicide, covid 19, emergency

## Abstract

**Introduction:**

COVID-19 pandemic is the most important health emergency of the 21st century. Since the high number of infected people and as there is still no specific therapy worldwide, the pandemic has been countered through the application of prevention measures based on social distancing and home isolation. These elements are known risk factors for the development of various psychiatric conditions. From a forensic point of view, these pathologies are related to a high suicide rate.

**Objectives:**

It is no coincidence that during the previous pandemics that have occurred in history there has been a significant increase in suicides. By this work, we therefore want to highlight the psychological consequences of a pandemic and the importance of preventive strategies.

**Methods:**

It is important to focus not only on physical well-being but also on the psychological aspects that the pandemic produces in the daily life of each individual

**Results:**

If the infecting agent causes the death of millions of people around the world, the socio-economic context that is created indirectly determines as many deaths.

**Conclusions:**

Therefore it is necessary to underline how it is advisable to implement preventive measures in order to significantly reduce deaths from suicide, a problem with an important impact in the social and forensic fields.

**Disclosure:**

No significant relationships.

